# Salicylic acid enhances cell growth, fatty acid and astaxanthin production in heterotrophic *Chromochloris zofingiensis* without reactive oxygen species elevation

**DOI:** 10.1186/s13068-023-02449-2

**Published:** 2024-01-03

**Authors:** Xinwei Zhang, Zhao Zhang, Yanmei Peng, Yushu Zhang, Qingyang Li, Dongzhe Sun

**Affiliations:** 1 Ministry of Education Key Laboratory of Molecular and Cellular Biology, Hebei Collaborative Innovation Center for Eco-Environment, Hebei Research Center of the Basic Discipline of Cell Biology, College of Life Sciences, Hebei Normal University, Shijiazhuang, 050024 China; 2 School of Life Sciences, Hebei University, Baoding, 071000 China; 3 Institute of Life Sciences and Green Development, Hebei University, Baoding, 071000 China

**Keywords:** *Chromochloris zofingiensis*, Salicylic acid, Phytohormone, Total fatty acids, Astaxanthin, Reactive oxygen species

## Abstract

**Background:**

The induction of lipid and astaxanthin accumulation in microalgae is often achieved through abiotic stress. However, this approach usually leads to oxidative stress, which results in relatively low growth rate. Phytohormones, as important small molecule signaling substances, not only affect the growth and metabolism of microalgae but also influence the intracellular reactive oxygen species level. This study aimed to screen phytohormones that could promote the fatty acids and astaxanthin yield of heterotrophic *Chromochloris zofingiensis* without causing oxidative damage, and further investigate the underlying mechanisms.

**Results:**

In the present study, among all the selected phytohormones, the addition of exogenous salicylic acid (SA) could effectively promote cell growth along with the yield of total fatty acids (TFA) and astaxanthin in heterotrophic *C. zofingiensis*. Notably, the highest yields of TFA and astaxanthin were achieved at 100 μM SA, 43% and 97.2% higher compared with the control, respectively. Interestingly, the intracellular reactive oxygen species (ROS) levels, which are usually increased with elevated TFA content under abiotic stresses, were significantly decreased by SA treatment. Comparative transcriptome analysis unveiled significant alterations in overall carbon metabolism by SA. Specifically, the upregulation of fatty acid synthesis pathway, upregulation of β-carotene-4-ketolase (BKT) in carotenoid synthesis aligned with biochemical findings. Weighted gene co-expression network analysis highlighted ABC transporters and GTF2B-like transcription factor as potential key regulators.

**Conclusion:**

This study found that salicylic acid can serve as an effective regulator to promote the celling growth and accumulation of fatty acids and astaxanthin in heterotrophic *C. zofingiensis* without ROS elevation, which provides a promising approach for heterotrophic production of TFA and astaxanthin without growth inhibition.

**Graphical Abstract:**

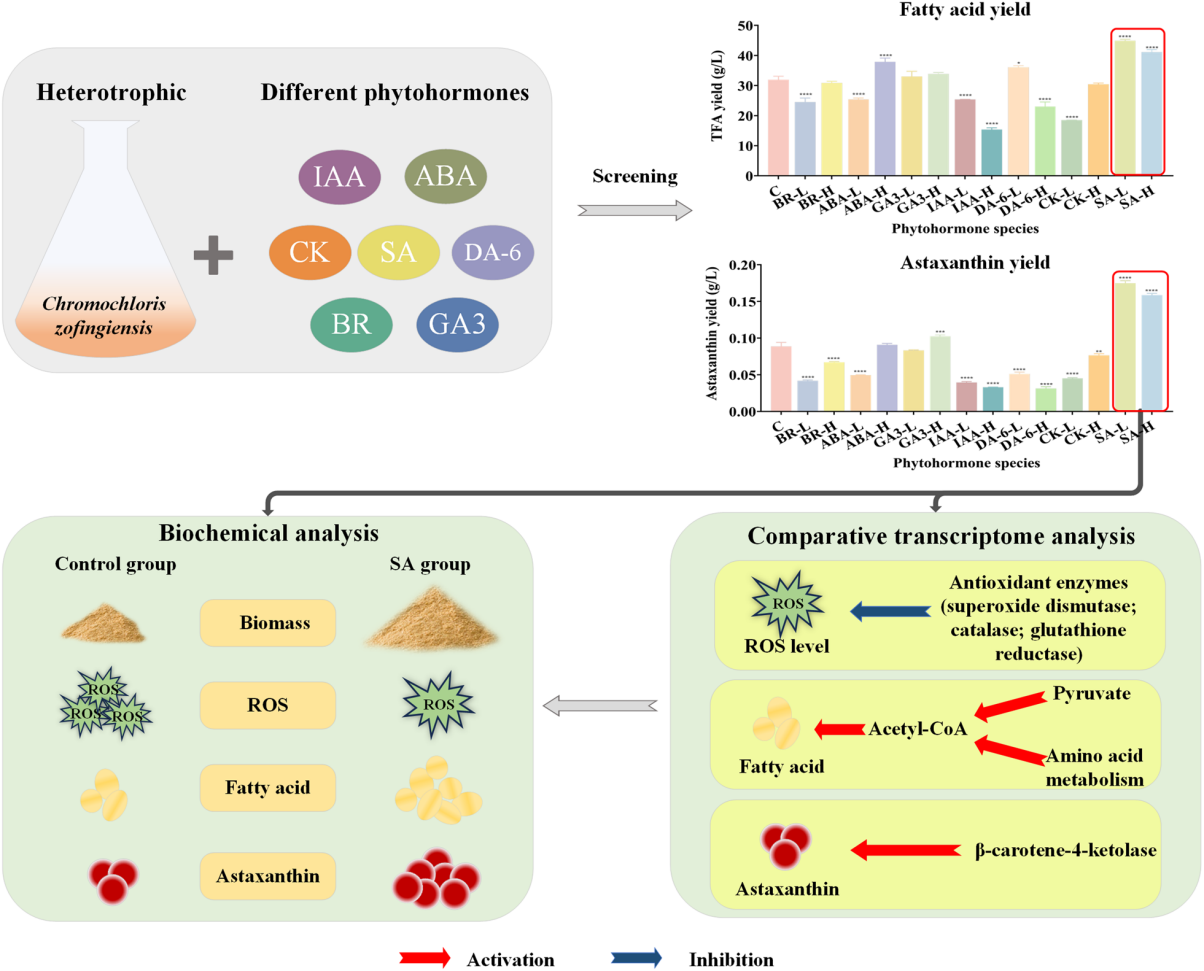

**Supplementary Information:**

The online version contains supplementary material available at 10.1186/s13068-023-02449-2.

## Background

Microalgae, widely distributed as single-celled microorganisms, constitute a crucial group of primary producers in the global ecosystem [1]. With the ability to synthesize proteins, pigments, lipids and other functional compounds, it can also be artificially utilized for production [2]. Particularly, the high content of lipid in oil-producing microalgae offering a promising source for biodiesel production. These microalgae exhibit a theoretical oil production potential 10 to 100-fold higher than that of terrestrial plants, positioning them as a key player in third-generation bioenergy [3, 4]. Furthermore, the generation of valuable carotenoids like β-carotene and astaxanthin has also been done using microalgae [5]. Microalgae can be cultivated through three trophic modes, including photoautotrophy, heterotrophy and mixotrophy. Among these, heterotrophic fermentation stands out as a preferred nutritional paradigm to produce high-value products. This approach circumvents the slow growth rate associated with autotrophic growth and the difficulty posed by attaining optimal light intensity in mixotrophic high-density cultures.

*C. zofingiensis* is considered as an emerging model strain with a well-defined genetic background, and could also be used for the co-production of lipids and astaxanthin via heterotrophic fermentation [6]. The highest biomass of *C. zofingiensis* was achieved through fed-batch fermentation at 98 g/L [7]. However, when compared with other microalgae, the lipid and astaxanthin contents in heterotrophic culture still have significant room for improvement. The usual methods for encouraging the formation of secondary metabolites in microalgae include altering cultivation conditions, imposing abiotic stress, and introducing chemical inducers. Notably, abiotic stress, encompassing factors like intense light, excessive salinity, nutrient deficiency, and heavy metal, is a frequently utilized induction technique [3]. Substantial attention has garnered to the effects of diverse stress situations on total fatty acids (TFA) and carotenoid accumulation in *C. zofingiensis,* and their metabolic mechanisms. For example, nitrogen deficiency adversely affects growth while increasing fatty acid and carotenoid accumulation by enhancing the expression of key enzymes in fatty acid and astaxanthin synthesis pathways, including acetyl-CoA carboxylase (ACCase), phytoene synthase (PSY), and beta-carotene 4-ketolase (BKT) [8, 9]. Besides, elevated reactive oxygen species (ROS) often accompany stress, inflicting oxidative damage compromises efficient product accumulation escalates production costs. These have led to limitations in the commercial application of microalgae, impeding its progress [10]. Therefore, researchers are actively seeking methods to enhance the accumulation of astaxanthin, fatty acid, and other compounds, while safeguarding growth and preventing oxidative damage.

Phytohormone serves as crucial signaling molecules, encompassing both natural compounds and artificially synthesized variants [11]. These molecules elicit profound physiological responses even at extremely low concentrations, exerting regulatory role over the entirety of plant life processes [12]. Notably, previous research has hinted at the evolutionary link between the phytohormone system in higher plants and the metabolic system inherent in microalgae [13]. Thus, it is speculated that the introduction of phytohormone into microalgae culture systems could potentially exert an impact on their growth and metabolism. This supposition finds support in various lines of evidence, with previous findings showcasing that the exogenous application of phytohormone can alleviate abiotic stress on microalgae and promote the accumulation of specific secondary metabolites. For instance, in the case of autotrophic *Dunaliella tertiolecta* under salt stress, the introduction of auxin resulted in a significant increased biomass accumulation by 40% and lipid content by 46% [14]. Additionally, addition of diethyl aminoethyl hexanoate to autotrophic cultures of *Chlorella sorokiniana* under nitrogen-deficient conditions resulted in a 43% and 84% enhance in biomass and lipid productivity, respectively [15]. However, these studies have been conducted under photoautotrophic condition, with limited exploration into the underlying mechanisms governing the effects of phytohormone on growth, TFA synthesis and carotenoid production during heterotrophic cultivation.

In this study, *C. zofingiensis* was employed as the model organism to systematically investigate potential phytohormone capable of promoting the accumulation phytohormone of TFA and carotenoid under heterotrophic condition. The research unveiled that salicylic acid (SA) holds the capability to increase both biomass production and the level of TFA and astaxanthin. Subsequent analyses were carried out to assess the impact of various concentrations of SA on growth dynamics, intracellular ROS levels, TFA and carotenoid contents. Furthermore, a comparative transcriptome analysis was conducted to unravel the underlying mechanism through which SA regulates fatty acid and carotenoid content. This study not only contributes novel insights into the potential for heterotrophic production of these valuable compounds but also serves as a valuable reference for investigations delving into the broader effects of phytohormones on microalgae.

## Materials and methods

### Strains and culture conditions

The *Chromochloris zofingiensis* strain (ATCC30412), obtained from the American Type Culture Collection (Rockville, MD, USA), was employed in this experiment. The Kuhl medium [16], supplemented with 5 g/L glucose, served as the culture medium. For inoculation, 10% (v/v) of the medium's volume was used. The initial cell density was 18.55×10^7^ cells/mL. The incubation temperature was 25°C and 150 rpm. This process took place in the absence of light for a duration of 4 days. Subsequently, the activated algae were transferred with a 10% inoculation volume to another 500 mL flask containing 100 mL of the medium, and cultivated for 4 days to reach the logarithmic growth phase. The resulting seed solution was employed for subsequent cultivation experiments.

### Inducting *C. zofingiensis* with seven phytohormones

The seed solution was inoculated to a medium containing seven phytohormones at varying concentrations, respectively. The concentration of each phytohormone was set as follows: Indole-3-acetic acid (IAA) (45 μM, 90 μM); Gibberellic acid (GA3) (10 μM, 19 μM); Cytokinin (CK) (2.3 μM, 23 μM); Abscisic acid (ABA) (38 μM, 90 μM); Salicylic acid (SA) (50 μM, 100 μM, 250 μM, 500 μM); Brassinosteroid (BR) (0.01 μM, 0.42 μM); and Diethyl aminoethyl hexanoate (DA-6) (10 μM, 100 μM). Three parallel sets were established for each treatment group and maintained under conditions of 25 °C, 150 rpm, and light exclusion for a period of 3 days. Subsequently, the cells were harvested and utilized for the quantification of cell count, dry weight, fatty acid content, and carotenoid content.

### Measurement of dry weight and cell number

Quantification of cell number in *C. zofingiensis* used a hemocytometer. At the 72-h time point, 1 mL samples were collected and suitably diluted with distilled water. Subsequently, 10 μL of the diluted samples were carefully placed in the chambers of the hemocytometer under the cover glass. Cell enumeration was accomplished utilizing an optical microscope. Each assay was replicated three times. For the measurement of dry weight, 5 mL of the *C. zofingiensis* solution was collected at the 72-h mark. The supernatant was removed through centrifugation, followed by two washes with distilled water. The residue was then filtered onto pre-dried and weighed filter paper (Whatman, England, CAT No.1442-070). Subsequently, it was subjected to a vacuum drying oven set at 85 °C for a duration of 4 h to eliminate cellular water content. The discrepancy in weight before and after loading sample yielded the dry weight of the cells. All procedures were conducted in triplicate.

### Fatty acid analysis

Aliquot 20 mg lyophilized *C. zofingiensis* cells were added into a 5 mL glass vial with lid. Add 1 mL of toluene, along with 2 mL of a 1% (v/v) sulfuric acid dissolved in methanol. To prevent lipid oxidation, 0.05% of 2,6-di-tert-butyl-4-methylphenol was added to the sample. Additionally, introduce 0.5 mL of heptadecanoic acid (C17:0). Thoroughly mix the contents and then proceed with methylation at 85 ℃ for 2.5 h. During this process, ensure to shake the sample every 30 min to ensure comprehensive methylation. To separate the aqueous and organic layers, after cooling, 1 mL of 0.75% NaCl solution was added and mixed. Then, 2 mL of chromatographic grade hexane was added for extraction, subsequently transferring the organic phase to a centrifuge tube. Eliminate residual solvent through drying via a nitrogen evaporator. After nitrogen evaporation, incorporate 1 mL of chromatographic grade hexane into the solution, and the mixture can be filtered through an injector and filter head into a sample vial. For analysis, an Agilent 7890 A gas chromatograph (GC) equipped with a DB-23 capillary column (30 m × 0.25 mm × 0.25 µm, Agilent, Santa Clara, CA, USA) was employed. Peak retention time and area were recorded, and then calculated the TFA content using the peak area of the C17:0 standard. All assessments were conducted in triplicate.

### Carotenoid analysis

Twenty milligrams of lyophilized *C. zofingiensis* powder was weighed with weighing paper and an analytical balance. Then, transfer the powder into a mortar and proceed to grind, the cells of *C. zofingiensis* were thoroughly broken down. The resulting sample was then subjected to extraction with chromatographic acetone, well shaken and centrifuged at 4 °C for 5 min at 10,000 rpm. The residue from the samples underwent two additional extractions with chromatographic acetone until the residue turned colorless. The acetone extracts from all three extractions were pooled together. After removing excess solvent with a nitrogen blower, the pigment was solubilized in 1 mL of chromatographic acetone, and then strained through a 0.45 µm pore size organic phase filtration membrane into a brown sample vial. The samples were subsequently analyzed using a high-performance liquid chromatography instrument (Waters, Milford, MA, USA) equipped with a C18 column. Retention time and peak area were recorded, and concentrations of the samples were determined by fitting the peak areas to the regression equations derived from standard curves of different carotenoids. All experiments were performed in three biological replicates.

### Measurement of reactive oxygen levels

The measurement of ROS levels within the cells was conducted using the ROS assay kit (Beyoncé, China). 1 mL of algal solution was sampled at 0 h, 12 h, 24 h and 72 h, respectively. The algal cells were reaped by subjecting the solution to centrifugation at 12,000 rpm for 3 min. After centrifugation, the sample after supernatant removal was resuspended into 200 μL of fresh Kuhl medium containing 10 μM 2,7-dichlorodihydrofluorescein diethyl ester (DCFH-DA). Subsequent to thorough mixing, the above treatment solution was incubated at 25 °C for 20 min away from light, with intermittent shaking every 5 min. Following incubation, the samples were collected and centrifuged at 12,000 rpm for 3 min. The precipitate was re-suspended in 1 mL of fresh Kuhl medium, centrifuged, the supernatant was removed, and repeated this process three times, under the same conditions as above. Finally, the samples were resuspended in their original medium, and absorbance values at 520 nm were measured under 485 nm excitation light. All procedures were conducted in triplicate.

### Comparative transcriptome analysis

Fifty milliliters of algal solution were collected at 24 h, 48 h and 72 h, followed by centrifugation. Subsequently, the samples were rapidly frozen with liquid nitrogen, ground in a mortar and pestle, and then transferred into centrifuge tubes. The Trizol technique was used to extract the total RNA. Next, Oligo(dT) were utilized to specifically bind mRNA with a Poly A tail. The isolated mRNA was broken up by adding a lysis buffer. Reverse transcriptase was used to create the initial chain of the cDNA molecule utilizing mRNA as a templet, then the second strand, creating a stable double-stranded structure. Adapters were subsequently ligated to the double-stranded cDNA, followed by purification and fragmented selection. The selected fragments underwent PCR amplification, and after purification, the final library was obtained.

The second-generation sequencing technology, the Illumina NovaSeq6000 was employed. The obtained data were compared with the *C. zofingiensis* reference genome to obtain mapping data for transcript assembly and expression quantification, a process performed on HiSat2 software. Differential expression calculation of read count using DESeq2 software. Genes were considered differentially expressed when DEGseq < 0.001 and |log2FC|≥ 1. In addition, enrichment analyses including Gene Ontology (GO), Kyoto Encyclopedia of Genes and Genomes (KEGG) and weighted gene co-expression network analysis (WGCNA) were conducted to investigate functional sets, pathway sets, gene sets, and the interrelationships within the differential gene set. All tests were performed in triplicate. The RNA-seq data can be obtained from Genome Sequence Archive via the accession number CRA012329.

### Real-time quantitative PCR (RT-qPCR)

The cDNA was obtained by reverse transcription of the total RNA obtained using the PrimeScript™ IV 1st strand cDNA kit (TaKaRa, Japan), in accordance with the manufacturer's instructions. Nine genes were randomly selected and primers for subsequent RT-qPCR were designed using Primer Premier5 software (see Additional file 1: Table S1). The TB Green Premix Ex TaqTMII kit (TaKaRa, Japan) was used to perform quantitative PCR using the cDNA as a template. Moreover, the2^−ΔΔCt^ technique, a relative quantitative method, was used to analyze gene expression [17].

### Statistical analysis

Three biological replicates were used in each experiment. Data were evaluated using one-way analysis of variance (ANOVA) and displayed as mean standard deviation (SD). P < 0.05 was used to evaluate significance. The statistical studies were carried out with the aid of GraphPad Prism 9.0.0.

## Results and discussion

### Influence of various phytohormones on growth, fatty acid, and astaxanthin content of *C. zofingiensis*

**Fig. 1:**
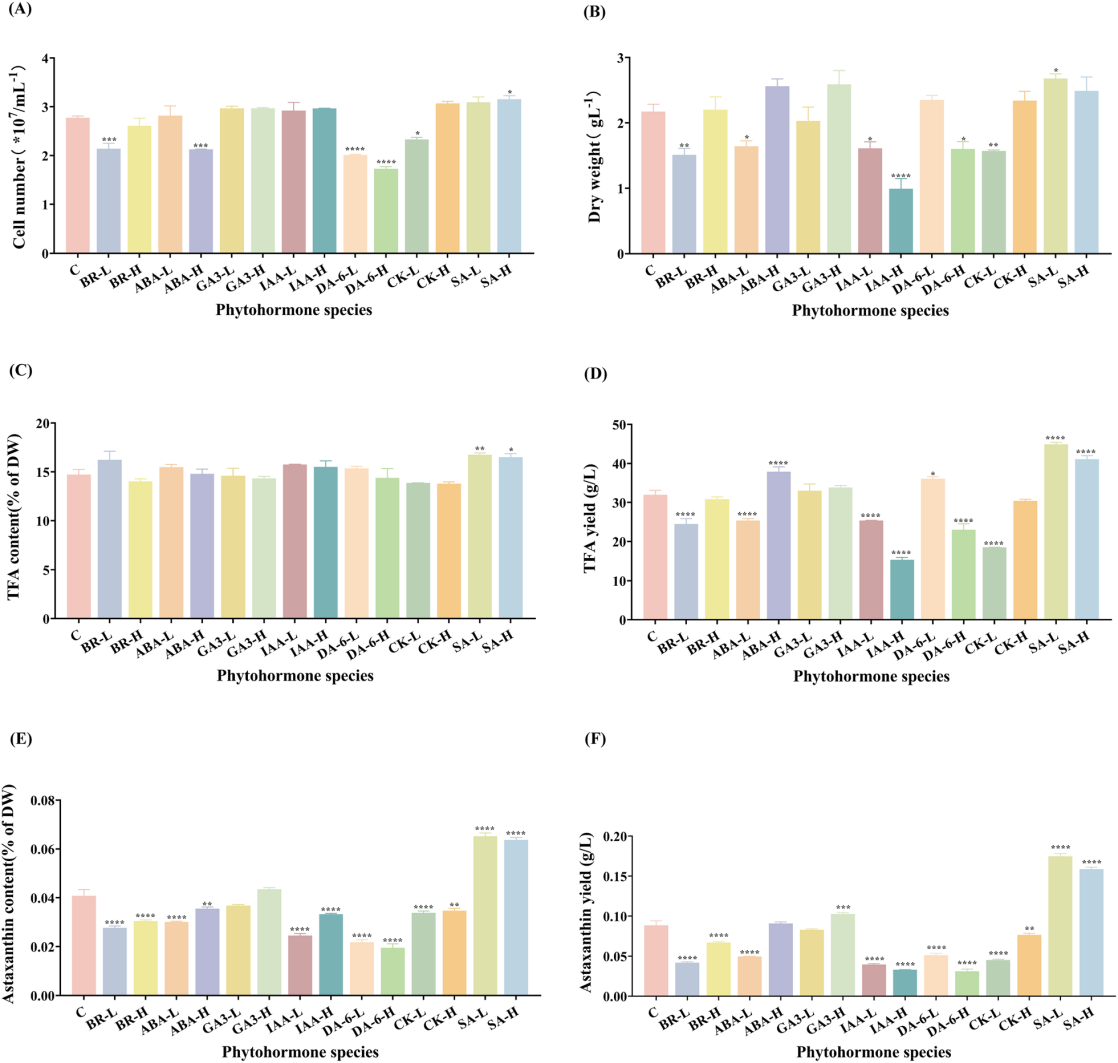
The effect of several phytohormones on the following characteristics of *C. zofingiensis*: cell number (**A**), dry weight (**B**), TFA content (**C**), TFA yield (**D**), astaxanthin content (**E**), and astaxanthin yield (**F**)*.* **C**: control group; BR-L: 0.01 μM BR group; BR-H: 0.42 μM BR group; ABA-L: 38 μM ABA group; ABA-H: 90 μM ABA group; GA3-L: 10 μM GA3 group; GA3-H: 19 μM GA3 group; IAA-L: 45 μM IAA group; IAA-H: 90 μM IAA group; DA-6-L: 10 μM DA-6 group; DA-6-H: 100 μM DA-6 group; CK-L: 2.3 μM CK group; CK-H: 23 μM CK group; SA-L: 100 μM SA group; SA-H: 500 μM SA group. Each experiment was carried out using three biological replicates, and the results are shown as mean ± SD. The statistical significance of the data was evaluated using a two-way repeated measures ANOVA; * denotes significant difference from the control group (p ≤ 0.05); ** indicates significant difference from the control group (p ≤ 0.01); *** signifies significant difference from the control group (p ≤ 0.001); ****represents significant difference from the control group (p ≤ 0.0001)

To screen for phytohormones that promote the growth and secondary product accumulation of heterotrophic *C. zofingiensis*, the cell count, dry weight (DW), TFA, and astaxanthin content of *C. zofingiensis* under different phytohormone treatments were determined. As shown in Fig. 1A, it's evident that the cell numbers in groups treated with 0.01 μM BR, 90 μM ABA, 10 μM and 100 μM DA-6, and 2.3 μM CK considerably reduced in contrast to the control. Conversely, the group treated with 500 μM SA exhibited a significantly increase in cell number in contrast to the control. Other phytohormones had little to no effect on the heterotrophic *C. zofingiensis* cell number. The alteration in DW displayed a distinct pattern from the cell number changes. Figure 1B demonstrates a significant reduction in DW from the control group for the groups exposed to 0.01 μM BR, 38 μM ABA, 45 μM IAA, 90 μM IAA, 100 μM DA-6, and 2.3 μM CK, with decreases of 30.4%, 24.4%, 25.8%, 54.4%, 26.3%, and 27.6%, respectively. Conversely, the DW of the group treated with 100 μM SA-treated group was significantly increased compared to the control. The various phytohormones demonstrated a degree of regulatory influence on the growth of *C. zofingiensis* within the selected concentrations. Notably, SA and 19 μM GA3 exhibited growth-promoting effects.

As illustrated in Fig. 1C-D, the effects of several phytohormones on the TFA content and yield of *C. zofingiensis* were examined. Fig. 1C provides a summary of the TFA content resulting from the application of seven phytohormones, with only SA notably promoting TFA accumulation in *C. zofingiensis*. Because phytohormone has distinct impacts on *C. zofingiensis* development and TFA content, TFA yield was computed to facilitate a more comprehensive evaluation of these hormones. This calculation incorporates TFA content and DW and is succinctly summarized in Fig. 1D for a more thorough assessment of the impact of phytohormone. A reduction in TFA yield was observed in cases involving IAA, 100 μM DA-6, ABA, CK, as well as 0.01 μM BR. Even though the TFA content did not change much from the control group, this drop can be attributable to *C. zofingiensis's* growth suppression. Interestingly, even though 90 μM ABA and 10 μM DA-6 significantly decreased the cell number of *C. zofingiensis*, the DW and TFA content were higher contrasted to the comparison group. This phenomenon resulted in an increase in TFA yield, with values of 37.87 g/L and 36.09 g/L, respectively. These figures were 1.18 times and 1.13 times higher than the control group, respectively. Significantly, it's crucial to emphasize that SA not only stimulates growth but also increases the TFA content of *C. zofingiensis*, consequently enhancing TFA yield, which result in a 1.5-fold and a 1.3-fold enhancement comparing to the control with 100 μM SA and 500 μM SA, respectively.

Astaxanthin content and yield were evaluated in each treatment group to discern the phytohormones promoting astaxanthin accumulation during heterotrophic growth of *C. zofingiensis.* As illustrated in Fig. 1E-F, both the SA and high-concentration GA3 treatment groups exhibited elevated astaxanthin content and significantly higher astaxanthin yield compared to the control group. While GA3 led to a slight increase in astaxanthin content, the addition of 100 μM SA and 500 μM SA resulted in a notable 1.97-fold and 1.79-fold enhancement of astaxanthin yield, respectively, in comparison to the control group.

Based on the above findings, the SA treatment group showed the most significant effects on biomass of heterotrophic *C. zofingiensis*, as well as the accumulation of fatty acid and astaxanthin. To obtain a more comprehensive understanding of how SA impacts the growth of *C. zofingiensis* and its subsequent effects on TFA and carotenoid levels, two additional concentrations of 50 μM and 1000 μM were introduced. However, it's noteworthy that the concentration of 1000 μM SA exhibited a lethal effect on *C. zofingiensis*. Therefore, in the subsequent studies, SA concentrations of 50 μM, 100 μM, and 500 μM were employed.

### Effect of different salicylic acid concentrations on growth and reactive oxygen species level in *C. zofingiensis*

**Fig. 2:**
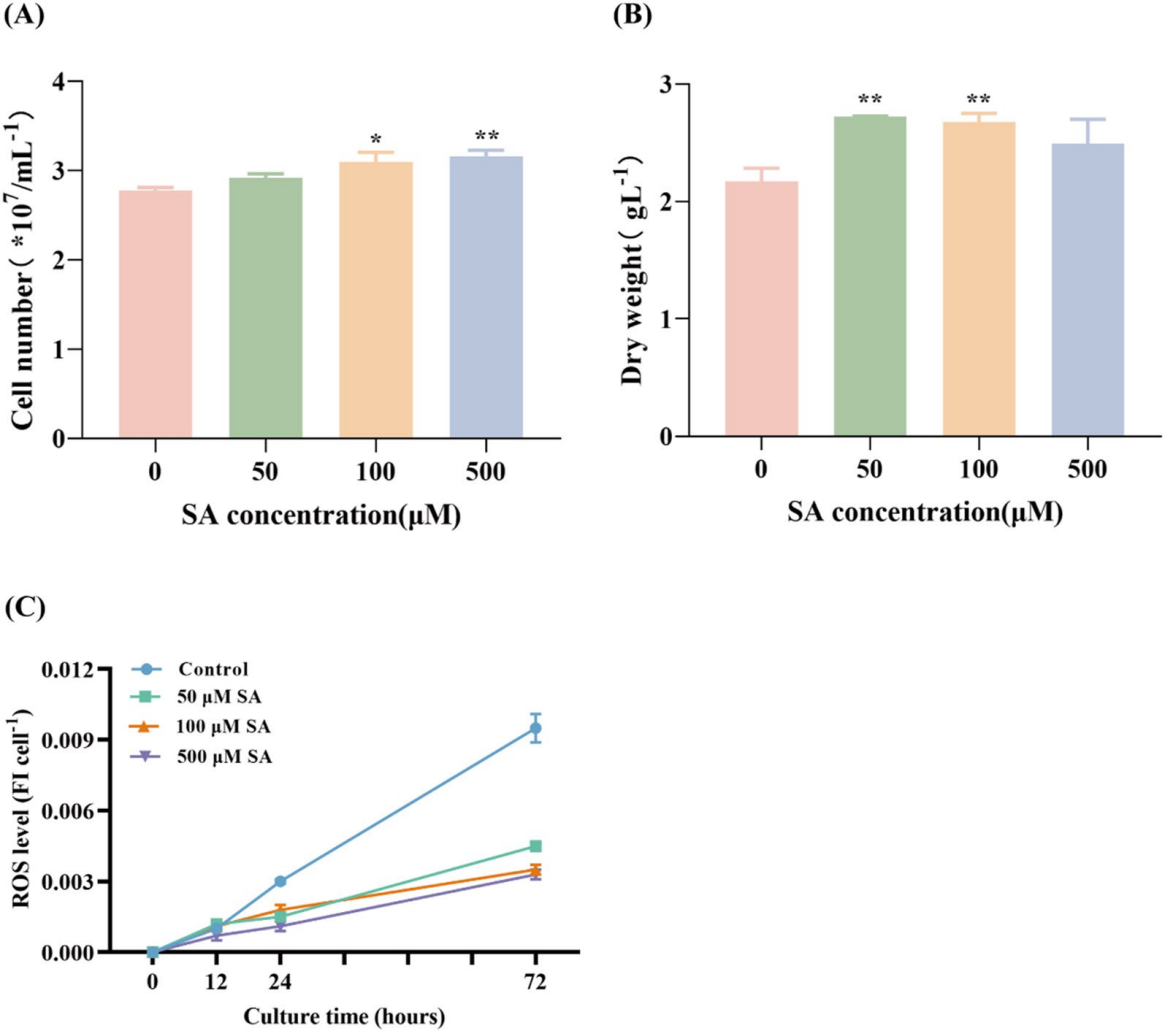
The effect of various concentrations of SA on cell number (**A**), dry weight (**B**) and ROS level (**C**) in *C. zofingiensis*. Each experiment was carried out using three biological replicates, and the results are shown as mean ± SD. The statistical significance of the data was evaluated using a two-way repeated measures ANOVA; * denotes significant difference from the control group (p ≤ 0.05); ** indicate significant difference from the control group (p ≤ 0.01)

To further investigate the effect of several concentrations of SA on *C. zofingiensis* biomass, cell number and DW were evaluated of *C. zofingiensis* during 3 days of heterotrophic cultivation. As illustrated in Fig. 2A, concentrations of SA ranging from 50 to 500 μM demonstrated a growth-promoting effect on *C. zofingiensis*. Specifically, the cell numbers of the 50 μM, 100 μM, and 500 μM SA treatment groups were 2.91 × 10^7^ cells/mL, 3.09 × 10^7^ cells/mL and 3.15 × 10^7^ cells/mL, respectively. The values exceeded those of the control group by 1.05 times, 1.11 times, and 1.17 times, respectively. Notably, the promotion effect on cell numbers increased with the rise in SA concentration. Contrastingly, Fig. 2B highlighted a different trend between DW and cell number. The DW exceeded than that of the control group under 50 μM, 100 μM, and 500 μM SA treatments, at 25%, 23%, and 14.7% higher levels, respectively. This suggests that low concentrations of SA have a more pronounced promoting influence on *C. zofingiensis* DW in comparison to high concentrations. In summary, the application of exogenous SA significantly influences *C. zofingiensis* growth, displaying a marked promotional effect. Furthermore, the lower concentration SA treatment group exhibited enhanced accumulation of intracellular substances in *C. zofingiensis* cells. Similarly, Czerpak reported a 1.4-fold increase in the cell number of autotrophic *Chlorella vulgaris* after 12 days of exposure to 100 μM SA [18]. Additionally, Fu observed an approximate 25% rise in cell density in *Chlorella regularis* with a treatment of 50 μg/L SA [19].

Cellular oxidative stress is typically quantified using ROS levels. ROS are natural byproducts of cellular metabolism, participating in intracellular signaling and regulation. They are essential for a number of biological functions, including the cell cycle, gene expression, and preserving homeostasis [20]. To investigate the impact of SA on ROS levels in *C. zofingiensis*, ROS levels were measured in groups treated with three different SA concentrations at 0 h, 12 h, 24 h, and 72 h. Figure 2C demonstrates that after 12 h, the ROS levels in all three SA-treated groups were noteworthy less than those in the control group, with the most notable change observed at 72 h. Exogenous SA used has been shown in prior investigations to result in a reduction in ROS levels [21], which is consistent with this research. This implies that SA effectively mitigates intracellular ROS accumulation in *C. zofingiensis*.

### Effect of different salicylic acid concentrations on fatty acid accumulation in *C. zofingiensis*

**Fig. 3:**
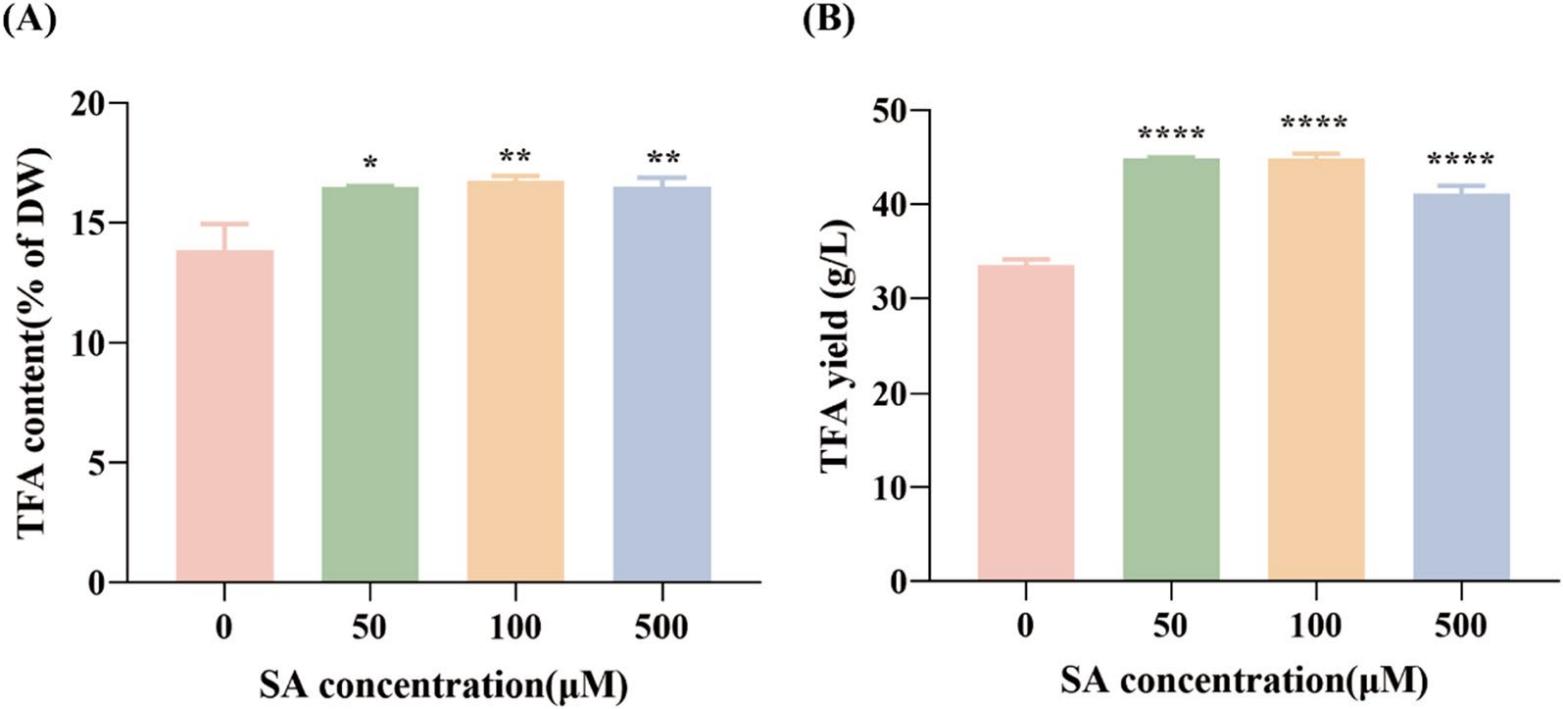
The effect of different concentrations of SA on TFA content and TFA yield (**B**) in *C. zofingiensis* (**A**). Each experiment was carried out using three biological replicates, and the results are shown as mean ± SD. The statistical significance of the data was evaluated using a two-way repeated measures ANOVA; * denotes significant difference from the control group (*p* ≤ 0.05); ** indicate significant difference from the control group (*p* ≤ 0.01); *** signifies significant difference from the control group (*p* ≤ 0.001); ****represents significant difference from the control group (*p* ≤ 0.0001)

Fatty acid serves as essential energy reserves in *C. zofingiensis*. In this experiment, after 72 h of culture, the TFA content and yield of each SA-treated group were assessed. As illustrated in Fig. 3A, the total fatty acids (TFA) content in the groups treated with 50 μM, 100 μM, and 500 μM SA was 16.49%, 16.75%, and 16.51% of the dry weight (DW), respectively. These values corresponded to 1.19 times, 1.21 times, and 1.19 times increments contrast to the control group. Evidently, SA concentrations ranging from 50 to 500 μM stimulate the accumulation of TFA within *C. zofingiensis* cells. TFA yield, calculated based on TFA content and DW, mirrored a similar trend, as depicted in Fig. 3B. Specifically, as contrasted with the control group, the groups treated with 50 μM, 100 μM, and 500 μM SA showed increases in TFA output of 42.9%, 43.0%, and 30.9%, respectively. These outcomes not only signify that SA stimulates TFA accumulation in *C. zofingiensis* cells but also enhances TFA yield by concurrently bolstering DW. Traditionally, stress factors including high light intensity, high salinity, and nitrogen deprivation are used to enhance the formation of fatty acid in microalgae. However, such stress conditions often induce cellular oxidative damage, impeding microalgae growth and yielding low fatty acid yield, which escalates production costs. For example, Yang et al.'s research showed that heightened salinity stress elevated microalgae fatty acid accumulation while inhibiting microalgae growth [22]. Anitha demonstrated that elevated temperature and salt coupling, as well as nitrogen deficiency and salt coupling, elevated the total lipid content of *Dunaliella* sp., but led to biomass reduction [23]. On the contrary, this study indicated that exogenous SA introduction did not elevate ROS levels. Furthermore, it promoted the synthesis and accumulation of fatty acid without impairing, and potentially enhancing *C. zofingiensis* growth (as shown in the Fig. 2).

The impact of distinct SA concentrations extends beyond TFA accumulation, encompassing alterations in the fatty acid composition of *C. zofingiensis*. The introduction of exogenous SA yielded significant shifts in the composition of TFA, as depicted in Table 1. Notably, the ratio of C18:1 in the TFA increased noticeably with the addition of exogenous SA, accompanied by a marked reduction in the percentage of C18:3. This effect was particularly pronounced when SA concentration escalated to 500 μM, causing the C18:1 content to increase from 30.11% to 33.04% of the TFA, while simultaneously causing the C18:3 content to decrease from 12.01% to 10.38% of the TFA. Analyzing changes in saturated fatty acids (SFAs), monounsaturated fatty acids (MUFAs), and polyunsaturated fatty acids (PUFAs), it becomes evident that the percentage of MUFAs increases from 30.11% to 33.04% as the SA concentration escalates to 500 μM. Conversely, the proportions of SFAs and PUFAs exhibit minor declines. However, it is important to note that the mechanisms through which SA regulates the fatty acid proportions in *C. zofingiensis* warrant further investigation.

**Table 1:** Fatty acid profile (%) variation in *C. zofingiensis* under different SA concentrations^a^

Fatty Acid	Control	50 μM SA	100 μM SA	500 μM SA
C16:0	23.56 ± 0.03	22.73 ± 0.14^*^	22.68 ± 0.06^*^	22.44 ± 0.30^*^
C16:3	2.05 ± 0.08	2.28 ± 0.10	2.07 ± 0.07	2.01 ± 0.03
C16:4	2.21 ± 0.08	2.20 ± 0.03	2.18 ± 0.03	2.44 ± 0.06^*^
C18:1	30.11 ± 0.13	32.61 ± 0.14^*^	32.82 ± 0.18^*^	33.04 ± 0.15^*^
C18:2	28.91 ± 0.12	28.83 ± 0.06	28.89 ± 0.12^*^	28.92 ± 0.09^*^
C18:3	12.01 ± 0.13	10.53 ± 0.06^*^	10.57 ± 0.17^*^	10.38 ± 0.07^*^
C18:4	1.16 ± 0.25	0.81 ± 0.03	0.79 ± 0.02	0.78 ± 0.01
SFAs	23.56 ± 0.03	22.73 ± 0.14^*^	22.68 ± 0.06^*^	22.44 ± 0.30^*^
MUFAs	30.11 ± 0.13	32.61 ± 0.14^*^	32.82 ± 0.18^*^	33.04 ± 0.15^*^
PUFAs	46.33 ± 0.16	44.65 ± 0.28	44.50 ± 0.23^*^	44.52 ± 0.16^*^

^a^Explanation of the notation for the fatty acid abbreviated: SFAs: The content of saturated fatty acids (% of TFA); MUFAs: The content of monounsaturated saturated fatty acids (% of TFA); PUFAs: The content of polyunsaturated fatty acids (% of TFA). * denotes significant difference from the control group (*p* ≤ 0.05)

### Effect of different salicylic acid concentrations on carotenoid content in *C. zofingiensis*

**Fig. 4:**
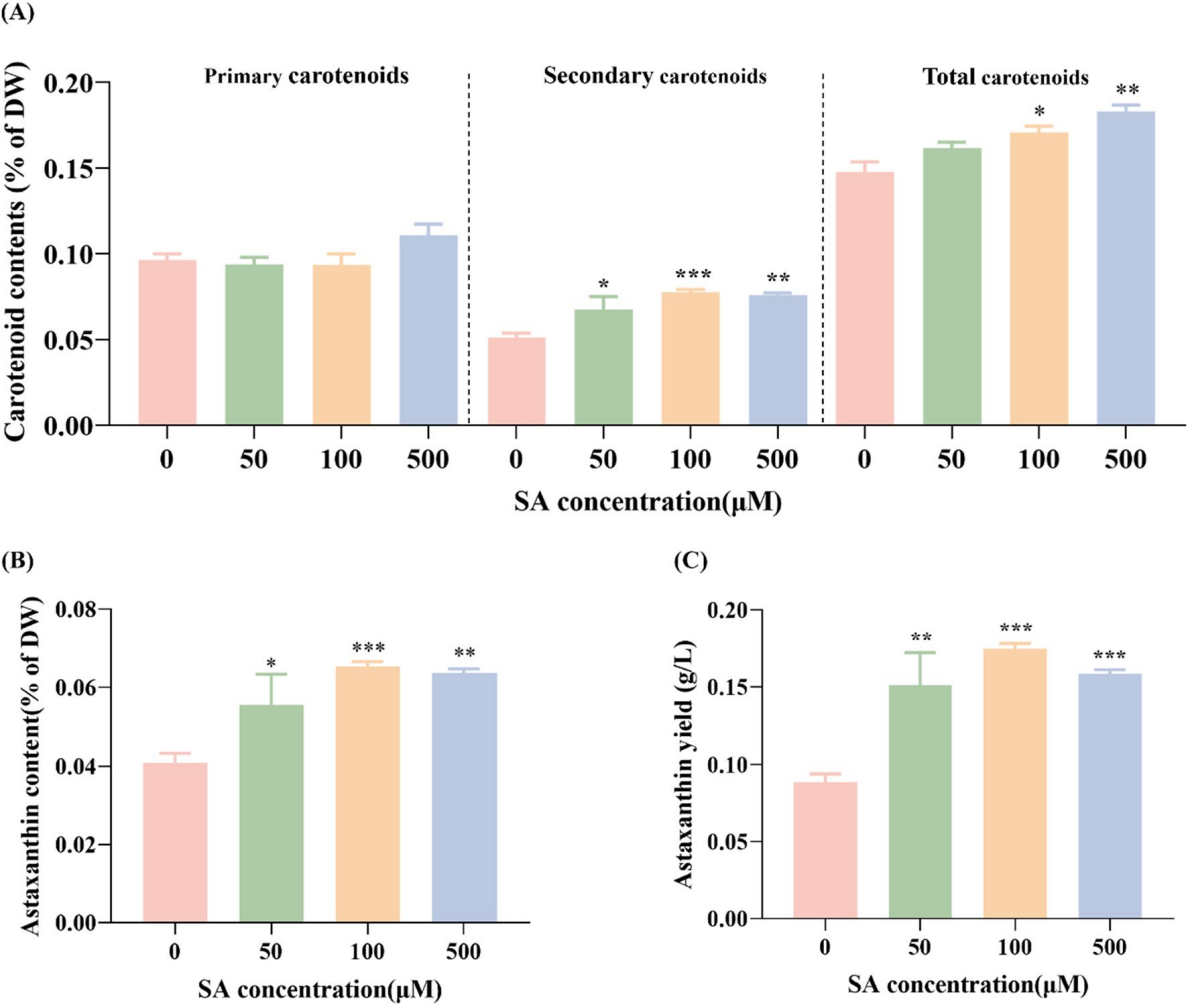
The effect of different concentrations of SA on carotenoid content (**A**), astaxanthin content (**B**) and astaxanthin yield (**C**) in *C. zofingiensis*. Each experiment was carried out using three biological replicates, and the results are shown as mean ± SD. The statistical significance of the data was evaluated using a two-way repeated measures ANOVA; * denotes significant difference from the control group (*p* ≤ 0.05); ** indicate significant difference from the control group (*p* ≤ 0.01); *** signifies significant difference from the control group (*p* ≤ 0.001); ****represents significant difference from the control group (*p* ≤ 0.0001)

To investigate the impact of SA on carotenoid content in *C. zofingiensis*, the carotenoid content and yield in *C. zofingiensis* were determined treated with varying SA concentrations. As shown in Fig. 4A, compared to the control group, the addition of exogenous SA caused a significant rise in the content of secondary carotenoids (including astaxanthin and adonixanthin). Consequently, there was a noticeable rise in the overall carotenoid content. Of particular interest, the content of astaxanthin exhibited a notable increase relative to the control group. This translated into enhanced astaxanthin yield, quantified at 0.151 g/L, 0.175 g/L, and 0.159 g/L for the 50 μM, 100 μM, and 500 μM SA-treated groups, respectively. These figures marked improvements of 70.6%, 97.2%, and 79.0% over the control group. This observation indicates that SA possesses the capability to promote astaxanthin accumulation within *C. zofingiensis*.

Similar with this study, in *Arabidopsis*, SA has been shown to elevate carotenoid content [24]. Moreover, SA has demonstrated the ability to enhance carotenoid content in *Chlorella pyrenoidosa* and *Haematococcus pluvialis* as well [25, 26]. According to this research, different SA concentrations led to an overall increase in total carotenoid content, particularly in secondary carotenoid. Given that β-carotene is an upstream precursor in astaxanthin synthesis, it can be hypothesized that SA might promote the conversion of β-carotene to astaxanthin. Previous research has highlighted the pivotal role of ROS in regulating astaxanthin synthesis. In photoautotrophic *Heamatococcus pluvialis* cells and heterotrophic *C. zofingiensis* cells, ROS has been demonstrated to increase astaxanthin accumulation [27, 28]. However, this experiment demonstrates that the upsurge in astaxanthin accumulation due to SA is concomitant with a reduction in ROS levels. This indicates that the mechanism through which SA promotes astaxanthin synthesis in *C. zofingiensis* might not be governed by ROS regulation.

### Comparative transcriptome analysis of heterotrophic *C. zofingiensis* under salicylic acid treatment

**Fig. 5:**
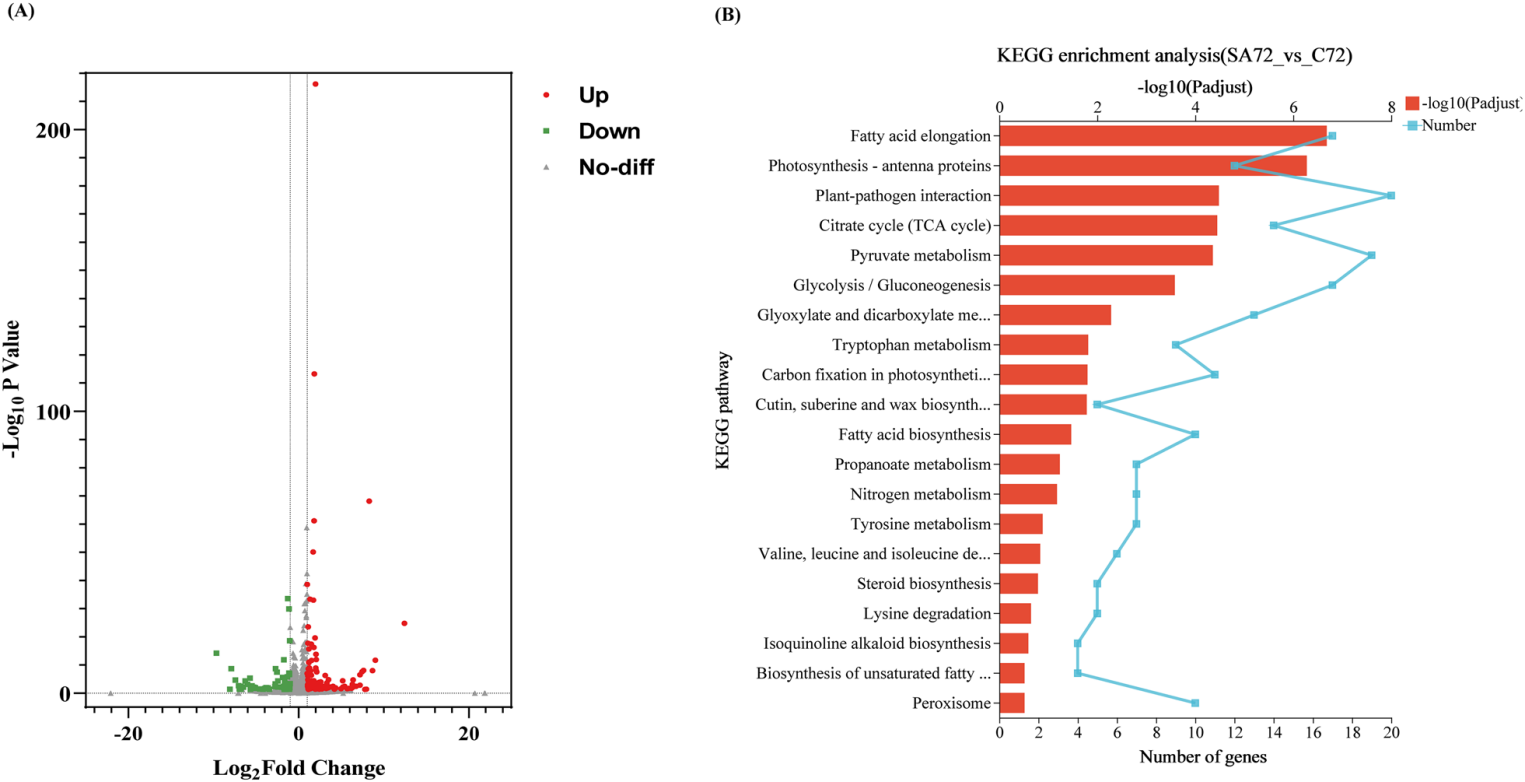
Transcriptome analysis. Genes analyzed for significantly upregulated, significantly downregulated, and insignificant changes in expression at 72 h (**A**). Pathway enrichment analysis results showing the significant metabolic pathways affected by SA treatment in *C. zofingiensis* (**B**)

To elucidate the mechanism of exogenous salicylic acid on the regulation of fatty acid and astaxanthin accumulation in *C. zofingiensis* under heterotrophic culture conditions, comparative transcriptome analysis was conducted on the samples. Since the fatty acid and astaxanthin content of the 100 μM SA-treated group was the highest of all concentrations, comparative transcriptome analyses were performed on the 100 μM SA-treated group at 24 h, 48 h, and 72 h, respectively, comparing it with the control group. Employing RNA-seq analysis, a total of 14,165 genes were detected, consisting of 13,873 known genes and 292 novel genes. Principal component analysis (PCA) was performed to verify the parallelism of three replications of all samples and the variability of the different treatment groups. The PCA exhibited a remarkable consistency among parallel groups and evident separation within the experimental group, affirming sound sample reproducibility and notable distinctions between treatment groups (see Additional file 1: Figure S1). In total, 3505 differentially expressed genes (DEGs) were identified and the number of upregulated and downregulated in each group was determined relative to controls (see Additional file 1: Table S2). Remarkably, as shown in Fig. 5A, the highest number of DEGs (2962 genes) were found at 72 h, indicating significant changes in cellular metabolism, with 114 strongly upregulated genes and 74 significantly downregulated genes.

To delve deeper into the specific metabolic pathways that play a role in these notable changes, KEGG pathway enrichment analysis was executed. Twenty different pathways were enriched as shown in the Fig. 5B, including those related to fatty acid metabolism such as fatty acid elongation, fatty acid biosynthesis, and biosynthesis of unsaturated fatty acids. Notably, carbon and nitrogen metabolism pathways such as citrate cycle, pyruvate metabolism, glycolysis/gluconeogenesis, and nitrogen metabolism pathways were also enriched. Similarly, amino acid metabolism pathways, including tryptophan metabolism, tyrosine metabolism, valine, leucine and isoleucine degradation, as well as lysine degradation, exhibited enrichment. These findings collectively suggest that SA significantly impacts intracellular protein, lipid and starch metabolism. In addition, the enrichment of peroxisomes in the analysis could potentially be linked to the modulation of ROS responses.

#### Analysis of acetyl-CoA origins and fatty acid synthesis pathways

**Fig. 6:**
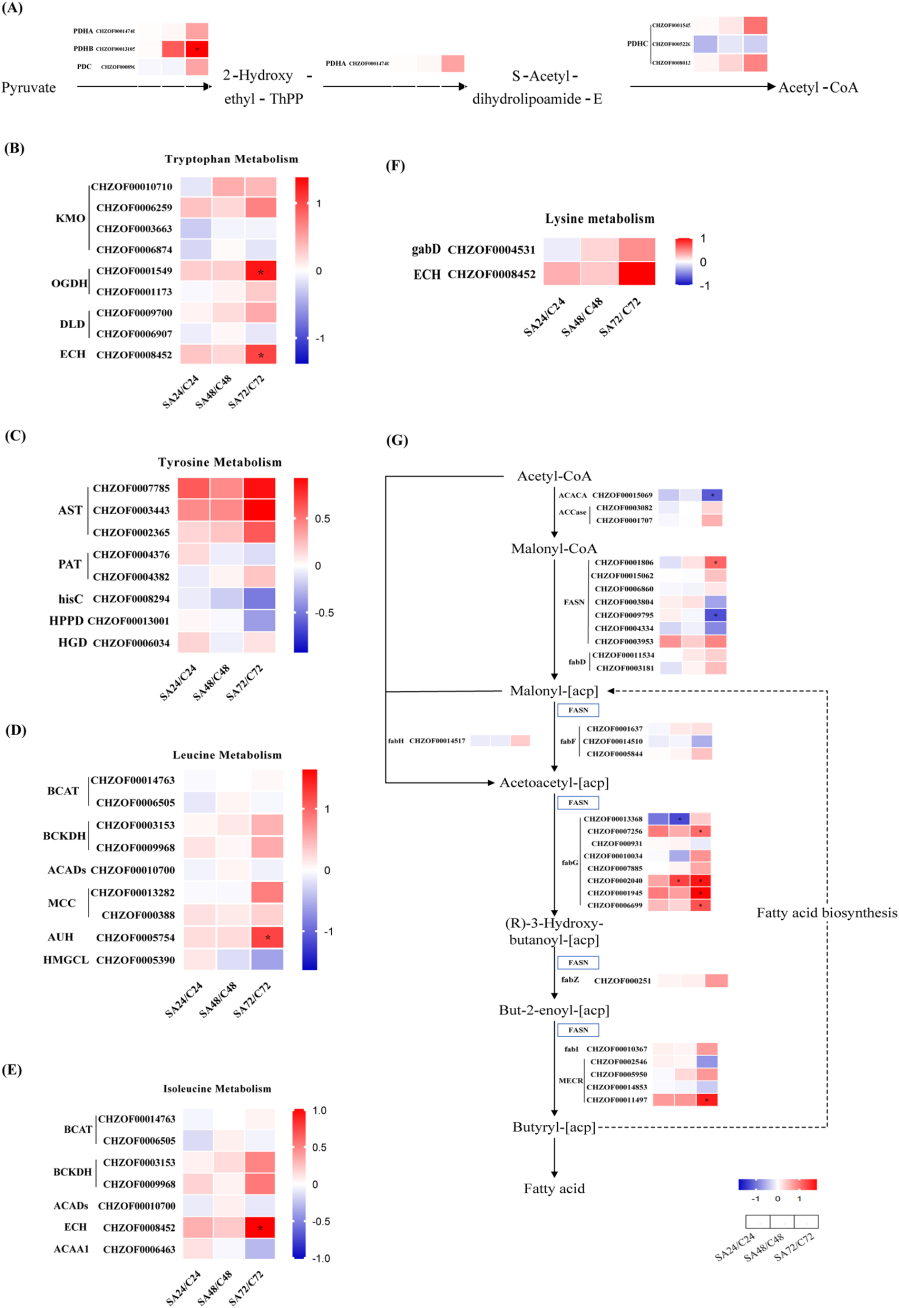
Expression profiles of genes involved in pyruvate metabolism (**A**), tryptophan metabolism (**B**), tyrosine metabolism (**C**), leucine metabolism (**D**), isoleucine metabolism (**E**), lysine metabolism (**F**) and fatty acid synthesis (**G**) pathway in *C. zofingiensis* based on transcriptomic analyses. PDHA: pyruvate dehydrogenase E1 component alpha subunit; PDHB: pyruvate dehydrogenase E1 component beta subunit; PDC: pyruvate decarboxylase; PDHC: pyruvate dehydrogenase E2 component; BCAT: branched-chain amino acid aminotransferase; BCKDH: 2-oxoisovalerate dehydrogenase; ACADs: acyl-CoA dehydrogenase; ECH: enoyl-CoA hydratase; ACAA1: acetyl-CoA acyltransferase 1; AST: aspartate aminotransferase; PAT: bifunctional aspartate aminotransferase and glutamate/aspartate-prephenate aminotransferase; hisC: histidinol-phosphate aminotransferase; HPPD: 4-hydroxyphenylpyruvate dioxygenase; HGD: homogentisate 1,2-dioxygenase; MCC: 3-methylcrotonyl-CoA carboxylase; AUH: methylglutaconyl-CoA hydratase; HMGCL: hydroxymethylglutaryl-CoA lyase; KMO: kynurenine 3-monooxygenase; OGDH: 2-oxoglutarate dehydrogenase; DLD: dihydrolipoyl dehydrogenase; gabD: uccinate-semialdehyde dehydrogenase / glutarate-semialdehyde dehydrogenase; ACACA: acetyl-CoA carboxylase1; ACCase: acetyl-CoA carboxylase; FASN: fatty acid synthase; fabD: [acyl-carrier-protein] S-malonyltransferase; fabF: 3-oxoacyl-[acyl-carrier-protein] synthase II; fabH: 3-oxoacyl-[acyl-carrier-protein] synthase III; fabG: 3-oxoacyl-[acyl-carrier protein] reductase; fabZ: 3-hydroxyacyl-[acyl-carrier-protein] dehydratase; fabI: enoyl-[acyl-carrier protein] reductase I; MECR: mitochondrial trans-2-enoyl-CoA reductase. (The expression levels of each gene were plotted on a heat map using the log2FC values in comparison to the control group; the * denoted a significant difference from the control group, p ≤ 0.05)

As an increase in cellular acetyl-CoA concentration can result in larger fatty acid accumulation, sufficient acetyl-CoA is necessary for the production of fatty acid in microalgae [29]. An imperative contributor to this synthesis process is pyruvate dehydrogenase (PDH), an enzyme catalyzing the conversion of pyruvate, a glycolysis product, into acetyl-CoA. Figure 6A highlights a notable upregulation in the expression of several PDH enzymes, particularly prominent at the 72-h mark, closely correlated with the observed TFA accumulation in our study. This phenomenon echoes the findings of Stao, underscoring the critical involvement of PDH in bolstering acetyl-CoA and fatty acid accumulation, as demonstrated in *Chlamydomonas sp. JSC4* [30]*.*

Amino acids, constituting the fundamental building blocks of living organisms, serve as another main source of acetyl-CoA through their degradation. Engaging in amino acid metabolism not only facilitates energy production, but also enables the synthesis of critical molecules like acetyl-CoA, while simultaneously maintaining cellular nutritional equilibrium [31]. In this study, the utilization of KEGG enrichment analysis unveiled metabolic pathways involved in the degradation of tryptophan, tyrosine, leucine, isoleucine, and lysine. Remarkably, all these amino acids can generate acetyl-CoA through their degradation pathways. Figure 6B-F delineates the examination of gene expression pertaining to enzymes within the pathway that contributes to acetyl-CoA production via the catabolism of tryptophan, tyrosine, leucine, isoleucine and lysine. The overall upregulation in the expression of enzymes central to these amino acid catabolic pathways was seen in Fig. 6B-F. Particularly noteworthy is the robust upregulation of enoyl-CoA hydratase (ECH), aspartate aminotransferase (AST), and methylglutaconyl-CoA hydratase (AUH) genes. ECH plays a vital role, catalyzing the hydration of crotonyl-CoA, generating (s)-3-Hydroxy-butanoyl-CoA, which subsequently reacts with acetoacetyl-CoA to form acetyl-CoA. An essential part of the metabolism of tryptophan, lysine, and isoleucine is played by this enzyme activities [32, 33]. Moreover, tyrosine is converted into 4-hydroxyphenylpyruvate through the transfer of amino groups from tyrosine to α-ketoglutarate, which is catalyzed by AST. AST is the rate-limiting enzyme for tyrosine catabolism [34]. AUH catalyze the 3-methylglutaryl-CoA into 3-hydroxy-3-methyl-glutaryl-CoA, which is a prerequisite for the generation of acetyl-CoA, a key step in the leucine catabolism [35]. Collectively, it becomes evident that the heightened breakdown of amino acids, prompted by the upregulated expression of specific genes, leads to a discernible escalation in acetyl-CoA synthesis. This phenomenon can be directly associated with the SA treatment applied in the study.

Fatty acid synthesis begins with the catalysis of acetyl-CoA to malonyl-CoA by acetyl-CoA carboxylase (ACCase). Then, the enzyme malonyl-CoA transacylase (MCAT) catalyzes the formation of acetyl-CoA into malonyl-ACP. Subsequently, a polymerisation reaction is carried out under the action of fatty acid synthase using acetyl coenzyme A and malonyl-ACP as substrates to extend the carbon chain. With each round of elongation, the carbon chain extends by two carbon atoms until a C16 or C18 carbon chain is formed [36]. As shown in the Fig. 6G, the majority of the genes related to fatty acid synthesis were elevated at 72 h. Among these genes, the heightened expression of ACCase and 3-Oxoacyl- (acyl carrier protein) reductase (fabG) emerged as particularly significant. The enzyme ACCase serves as the rate-limiting enzyme during the process of fatty acid synthesis and catalyzes the first step reaction. fabG emerges as a key enzyme, catalyzing the initial reduction reaction in fatty acid synthesis, mostly using NADPH as a coenzyme to facilitate the conversion of 3-ketolipid acyl ACP to 3-hydroxylipid acyl ACP.

In conclusion, the evidence indicates that SA exerts a promotive effect on acetyl-CoA production by controlling the gene expression of enzymes responsible for pyruvate metabolism and amino acid degradation pathways. An adequate supply of precursors for the synthesis of fatty acid is guaranteed by this regulatory activity. Furthermore, SA increases the expression of two crucial enzymes in the fatty acid synthesis pathway, fabG and ACCase, which has an additional enhancement on fatty acid formation.

#### Analysis of carotenoid synthesis pathway

Carotenoids are a category of terpenoids composed of isoprenoids. Isoprene pyrophosphate (IPP) and dimethylpropylene pyrophosphate (DMAPP), two of its key precursors, are produced through the mevalonate (MVA) and non-mevalonate (MEP) pathways [37]. The MEP route is the only one used in microalgae to synthesize IPP [38]. Subsequently, IPP and DMAPP were catalyzed by geranylgeranyl diphosphate synthase (GGPPS) and phytoene synthase (PSY) to synthesize phytoene; the production of lycopene was then catalyzed by phytoene desaturase (PDS) and ζ-carotene desaturase (ZDS). The production of various carotenoids is further facilitated by cyclization processes [39].

**Fig. 7:**
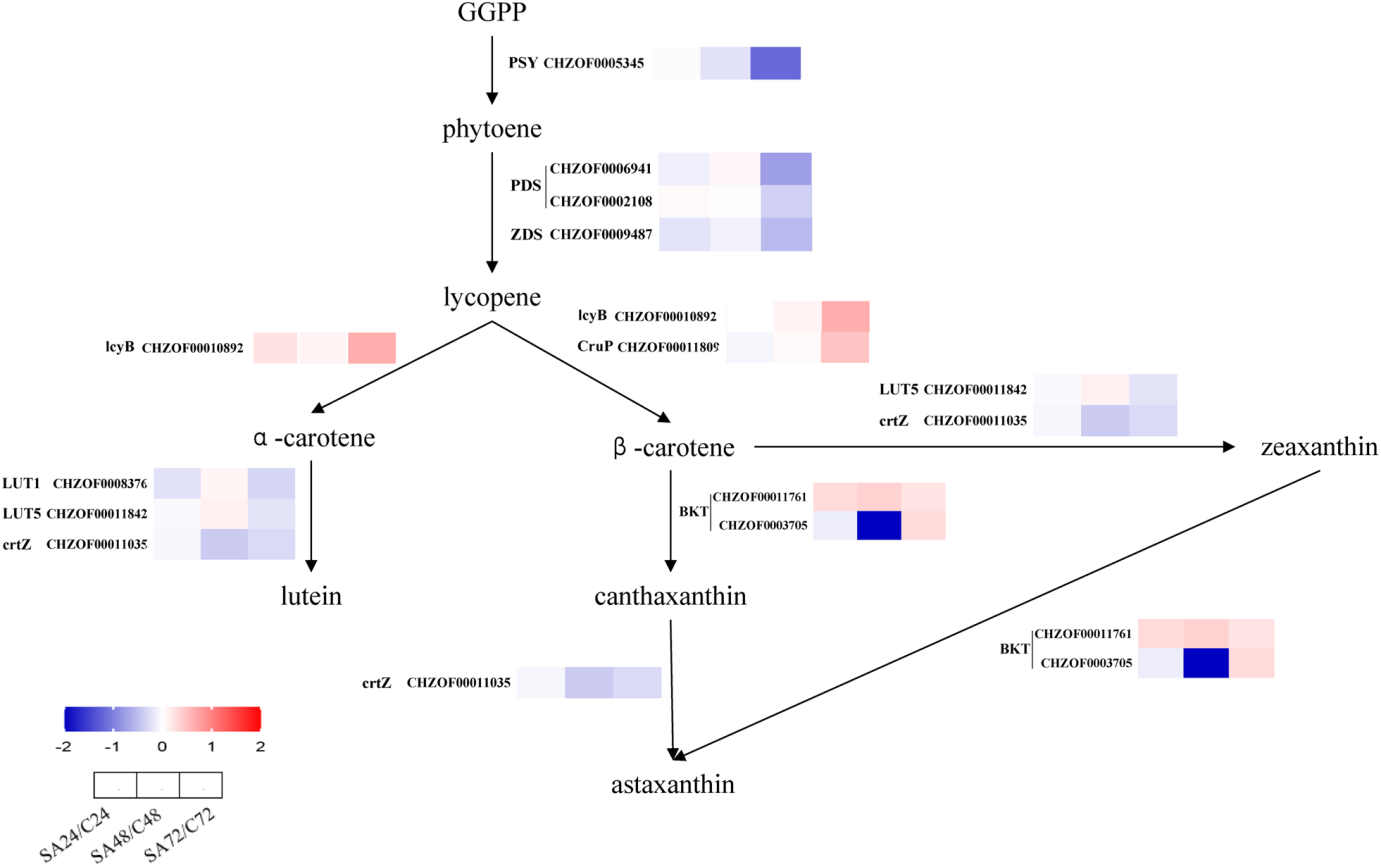
Expression profiles of genes involved in carotenoid synthesis in *C. zofingiensis* based on transcriptomic analyses. *PSY* phytoene synthase, *PDS* phytoene desaturase, *ZDS* zeta-carotene desaturase, *lcyB* lycopene beta-cyclase, *LUT1* carotenoid epsilon hydroxylase, *LUT5* beta-ring hydroxylase, *crtZ* beta-carotene 3-hydroxylase, *CruP* lycopene cyclase Crup, *BKT* beta-carotene 4-ketolase. (The log2FC values were utilized to create a heat map showing the expression levels of each gene compared to the control, the * was significantly different from the control group, *p* ≤ 0.05)

To investigate how SA influences the synthesis of carotenoids, a comprehensive analysis was conducted at the transcriptional level. As shown in Fig. 7, the gene expression pattern governing carotenoid biosynthesis proved to be intricate. Specifically, the genes responsible for the progression from GGPP to lycopene, α-carotene to lutein, β-carotene to zeaxanthin, and canthaxanthin to astaxanthin was downregulated, whereas the expression of genes directing the path from lycopene to α-carotene and β-carotene, β-carotene to canthaxanthin, and zeaxanthin to astaxanthin exhibited upregulation. In summary, the synthesis of lycopene, a precursor to primary carotenoid, experienced reduction, while the gene expression within the primary to secondary carotenoid pathway underwent a blend of upregulated and downregulated. Integrating these changes along with the alteration in primary and secondary carotenoid content, primary carotenoid content remained largely unchanged while astaxanthin content increased significantly. Therefore, this leads to the conclusion that SA promotes the expression of the β-carotene-4-ketolase (BKT) gene, favoring the conversion of β-carotene into astaxanthin.

#### Analysis of the antioxidant enzymes

**Fig. 8:**
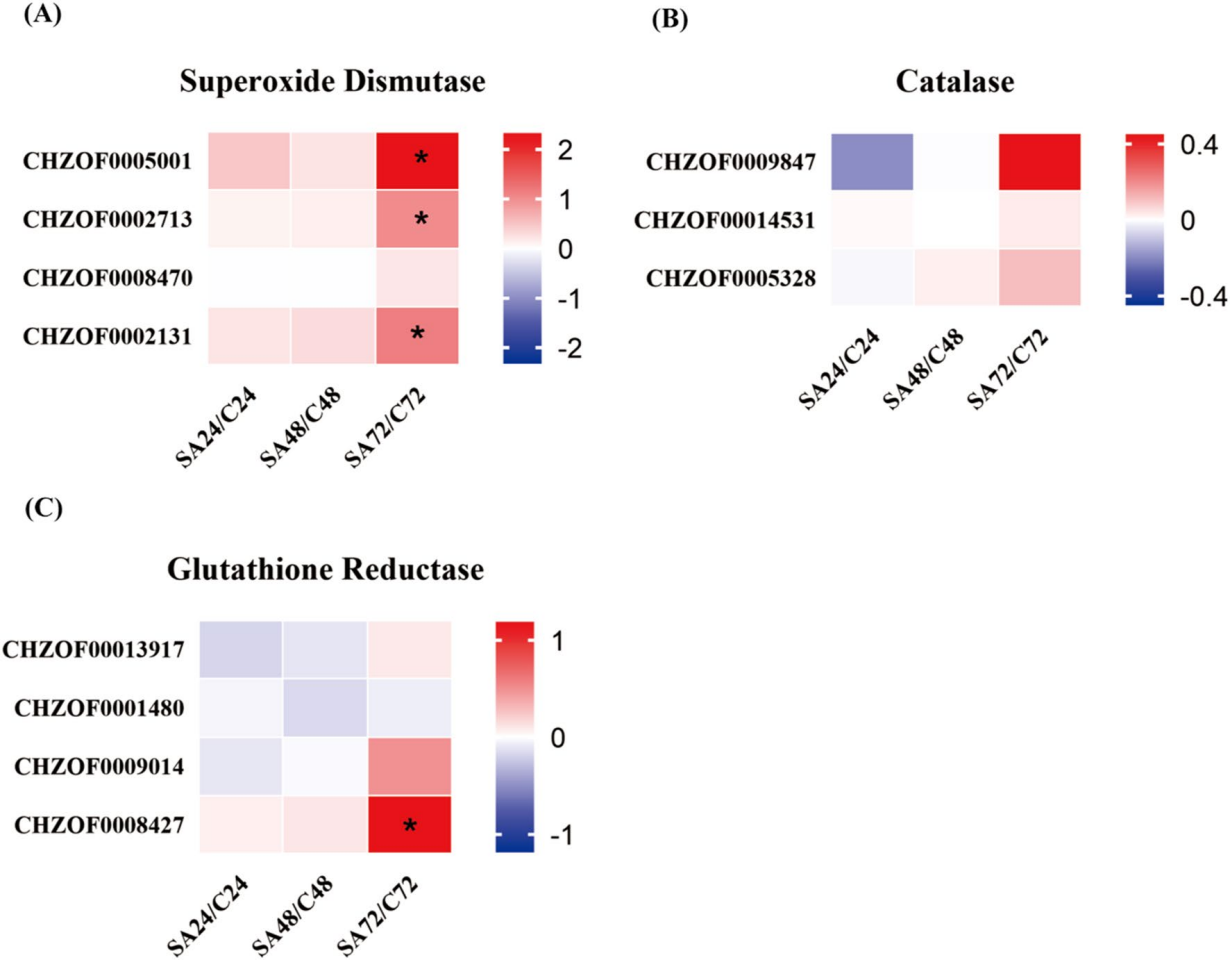
Analysis of the gene expression profiles of antioxidant enzymes in *C. zofingiensis*. (The log2FC values were utilized to create a heat map showing the expression levels of each gene compared to the control, the * was significantly different from the control group, *p* ≤ 0.05)

Antioxidant enzymes are essential, which to keep the equilibrium between oxidation and antioxidation inside organisms. They effectively convert excessive ROS and free radicals into less harmful compounds, thereby ensuring the appropriate ROS levels in the cells. Among these enzymes, superoxide dismutase (SOD) is a critical metalloenzyme found across microorganisms, plants and animals. It facilitates the catalytic disproportionation of superoxide anion radicals to generate oxygen and hydrogen peroxide [40]. Another key enzyme, catalase (CAT) operates predominantly within cell peroxisomes, catalyzing the decomposition of H_2_O_2_ into oxygen and water [41]. Glutathione reductase (GR) is a flavoprotein oxidoreductase widely found in eukaryotic and prokaryotic organisms that plays a critical role in glutathione redox reactions and reactive oxygen species scavenging [42]. To elucidate the association between variations in ROS levels and antioxidant enzymes, an analysis of gene expression related to SOD, CAT, and GR was conducted. As shown in the Fig. 8, the transcription of several antioxidant enzymes predominantly demonstrated an upregulated pattern, with notably elevated expression levels observed for SOD. Furthermore, there is a significant upregulation at 72 h. Taking into account the noteworthy reduction in ROS levels observed within the SA-treated group, a reasonable inference can be drawn that the application of SA works by enhancing the expression of antioxidant enzymes to reduce ROS levels.

#### Weighted gene co-expression network analysis

**Fig. 9:**
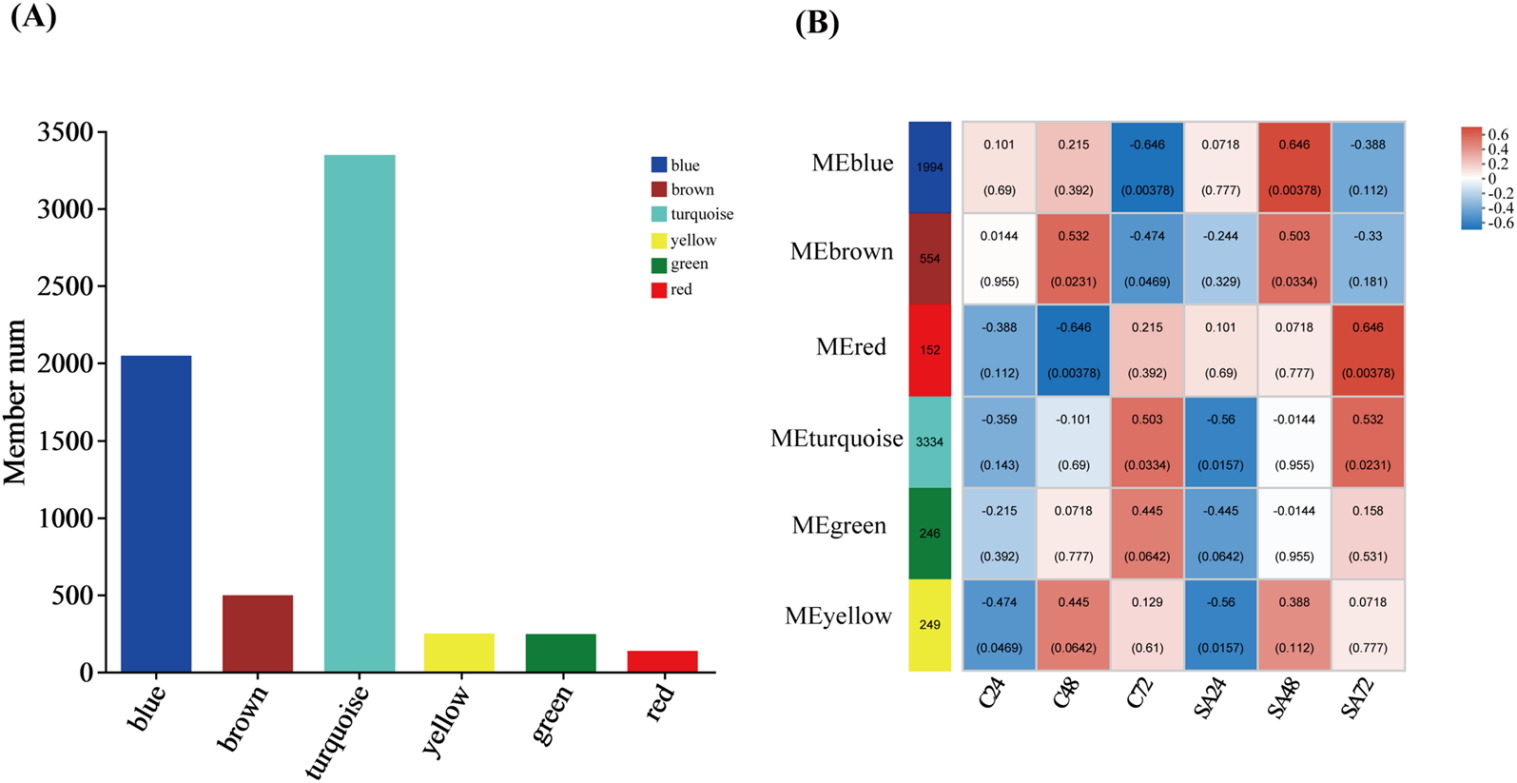
Number of genes in each clustered module (**A**). Correlation between module and trait (C24, C48, and C72, under without SA conditions for 24 h, 48 h, and 72 h, respectively; SA24, SA48, and SA72, under SA conditions for 24 h, 48 h, and 72 h, respectively)

The impact of SA on lipids and carotenoids can be determined by observing changes in gene expression. However, the specific mechanism by which SA impacts the synthesis of metabolites in *C. zofingiensis* cells remains unclear. Therefore, to group genes with comparable expression patterns and examine the connection between modules and certain traits or phenotypes, Weighted Gene Co-expression Network Analysis (WGCNA) was carried out. As shown in Fig. 9A, six modules were derived from co-expression analysis, with the turquoise module containing the highest gene count (3346 in total) and the red module containing the fewest genes (135 in total). As shown in Fig. 9B, the red module was favorably related with the SA-treated groups, according to the module-phenotype correlation analysis, while there was no significant trend in the other modules. The top 20 genes with the highest association were found by visual examination of 135 genes in the red module. As shown in Additional file 1: Table S3, it was observed that these genes were potentially associated with SA signal transduction, including ABC transporters, GTF2B-like transcription factor, as well as genes associated with fatty acid metabolism, amino acid metabolism and antioxidant enzymes.

ABC transporter proteins facilitate the energy-dependent transmembrane transport of substances between cells' interior and exterior using ATP hydrolysis [43]. According to research, SA can quickly activate the genes for ABC transporter proteins and boost the transmembrane efficiency of salicylic acid [44]. Transcription factors are specialized proteins that bind to specific regions of DNA known as cis-regulatory elements. Their main function is to positively or negatively regulate gene transcription in eukaryotes. GTF2B-like transcription factors are integral to the RNA polymerase I core factor complex, exerting vial roles in multiple transcription initiation steps. SA signaling is initiated through the recognition by the SA signaling receptor, SA-binding protein (SABP), subsequently regulating downstream gene expression via transcription factors [45]. The hypothesis suggests that the exogenous SA signaling pathway within *C. zofingiensis* could potentially undergo intracellular transmission facilitated by ABC transporter protein, subsequently regulates downstream gene expression via GTF2B-like transcription factors. However, this proposition is founded only based on genomic data, and its validation needs further investigation.

#### Real-time quantitative PCR validation of transcriptome data reliability

Nine genes were chosen at random for RT-qPCR validation to confirm the validity of the transcriptome data. These genes were distributed in the pathways of pyruvate metabolism, amino acid catabolism, fatty acid synthesis, and carotenoid synthesis. The observed variations in the levels of the nine randomly chosen genes compared to the control group were consistent with those in the transcriptome (see Additional file 1: Figure S2). This suggests that the transcriptome data can be considered trustworthy.

## Conclusions

In this study, among the selected phytohormones, SA demonstrated a significant capacity to increase the fatty acid and carotenoid content of *C. zofingiensis* under heterotrophic culture conditions without inducing oxidative damage. Comparative transcriptome analysis revealed that SA was able to upregulate the transcription of pyruvate metabolism, amino acid catabolism, fatty acid synthesis, and carotenoid synthesis pathways. WGCNA showed that ABC transporters and GTF2B-like transcription factor might be the key regulating factors. In summary, this study proposed that SA was an efficient regulator for enhancing the fatty acid and astaxanthin accumulation in heterotrophic *C. zofingiensis*, and delved into its underlying mechanisms.

### Supplementary Information

**Additional file1: Figure S1.** Principal component analysis (PCA). To validate both the reproducibility of the triplicate parallel samples and the differences among different treatment groups, we conducted principal component analysis (PCA). **Figure S2.** The differentially expressed genes detected by RT-qPCR at 24 h, 48 h and 72 h. **Table S1.** Real-Time Quantitative PCR primer sequences. To verify the reliability of the transcriptome data, nine genes were randomly selected for which primers were designed. **Table S2.** DEGs of different groups compared with control group cells at 24 h, 48 h and 72 h. **Table S3. **Genes annotated in the network analysis and describe.

**Additional file 2.** RNA-seq data. The "Gene ID", "Annotation", "Abbreviation", "TPM", "Log2fc", "Pvalue", "Padjust", "Regulate" and "Significant" of "Pyruvate metabolism", "Tryptophan metabolism", "Tyrosine metabolism", "Leucine metabolism", "Isoleucine metabolism", "Lysine metabolism", "Fatty acid synthesis", "Carotenoid synthesis" and "Antioxidant enzymes" pathways.

## Data Availability

All data generated or analyzed during this study are included in this published article [and its supplementary information files].

## Abbreviations

*C. zofingiensis*: *Chromochloris zofingiensis*
SA: Salicylic acid
TFA: Total fatty acids
ROS: Reactive oxygen species
TCA: Tricarboxylic acid
BKT: β-Carotene 4-ketolase
WGCNA: Weighted gene co-expression network
ACCase: Acetyl-CoA carboxylase
PSY: Phytoene synthase
IAA: Indole-3-acetic acid
GA3: Gibberellic acid
CK: Cytokinin
ABA: Abscisic acid
BR: Brassinosteroid
DA-6: Diethyl aminoethyl hexanoate
C17:0: Heptadecanoic acid
NaCl: Sodium chloride
DCFH-DA: 2′, 7′-Dichlorofluorescin diacetate
RNA: Ribonucleic acid
mRNA: Messenger ribonucleic acid
cDNA: Complementary deoxyribonucleic acid
GO: Gene ontology
KEGG: Kyoto encyclopedia of genes and genomes
RT-qPCR: Real-time quantitative polymerase chain reaction
SD: Standard deviation
ANOVA: One-way analysis of variance
DW: Dry weight; SFAs: Saturated fatty acids
MUFAs: Monounsaturated fatty acids
PUFAs: Polyunsaturated fatty acids
RNA-seq: RNA sequencing
PCA: Principal component analysis
PDHA: Pyruvate dehydrogenase E1 component alpha subunit
PDHB: Pyruvate dehydrogenase E1 component beta subunit
PDC: Pyruvate decarboxylase
PDHC: Pyruvate dehydrogenase E2 component
BCAT: Branched-chain amino acid aminotransferase
BCKDH: 2-Oxoisovalerate dehydrogenase
ACADs: Acyl-CoA dehydrogenase
ECH: Enoyl-CoA hydratase
ACAA1: Acetyl-CoA acyltransferase 1
AST: Aspartate aminotransferase
PAT: Bifunctional aspartate aminotransferase and glutamate/aspartate-prephenate aminotransferase
hisC: Histidinol-phosphate aminotransferase
HPPD: 4-Hydroxyphenylpyruvate dioxygenase
HGD: Homogentisate 1,2-dioxygenase
MCC: 3-Methylcrotonyl-CoA carboxylase
AUH: Methylglutaconyl-CoA hydratase
HMGCL: Hydroxymethylglutaryl-CoA lyase
KMO: Kynurenine 3-monooxygenase
OGDH: 2-Oxoglutarate dehydrogenase
DLD: Dihydrolipoyl dehydrogenase
gabD: Uccinate-semialdehyde dehydrogenase / glutarate-semialdehyde dehydrogenase; ACACA: Acetyl-CoA carboxylase1
FASN: Fatty acid synthase
fabD: [Acyl-carrier-protein] S-malonyltransferase
fabF: 3-Oxoacyl-[acyl-carrier-protein] synthase II
fabH: 3-Oxoacyl-[acyl-carrier-protein] synthase III
fabG: 3-Oxoacyl-[acyl-carrier protein] reductase
fabZ: 3-Hydroxyacyl-[acyl-carrier-protein] dehydratase
fabI: Enoyl-[acyl-carrier protein] reductase I
MECR: Mitochondrial trans-2-enoyl-CoA reductase
MCAT: Malonyl-CoA transacylase
PDH: Pyruvate dehydrogenase
NADPH: Nicotinamide adenine dinucleotide phosphate
MVA: Mevalonate
MEP: Non-mevalonate
IPP: Isopentenyl pyrophosphate
DMAPP: Dimethylallyl pyrophosphate
GGPPS: Geranylgeranyl diphosphate synthase
PDS: Phytoene desaturase
ZDS: Zeta-carotene desaturase
lcyB: Lycopene beta-cyclase
LUT1: Carotenoid epsilon hydroxylase
LUT5: Beta-ring hydroxylase
crtZ: Beta-carotene 3-hydroxylase
CruP: Lycopene cyclase Crup
SOD: Superoxide dismutase
CAT: Catalase
GR: Glutathione reductase
