# Supplementary material for: Salicylic acid enhances cell growth, fatty acid and astaxanthin production in heterotrophic Chromochloris zofingiensis without reactive oxygen species elevation
Source: Biotechnol Biofuels Bioprod. 2024 Jan 3;17:1. doi: 10.1186/s13068-023-02449-2 (PMC10765886; doi:10.1186/s13068-023-02449-2)
Supplement: Supplementary file 1 — Additional file1: Figure S1. Principal component analysis (PCA). To validate both the reproducibility of the triplicate parallel samples and the differences among different treatment groups, we conducted principal component analysis (PCA). Figure S2. The differentially expressed genes detected by RT-qPCR at 24 h, 48 h and 72 h. Table S1. Real-Time Quantitative PCR primer sequences. To verify the reliability of the transcriptome data, nine genes were randomly selected for which primers were designed. Table S2. DEGs of different groups compared with control group cells at 24 h, 48 h and 72 h. Table S3. Genes annotated in the network analysis and describe. [file 13068_2023_2449_MOESM1_ESM.docx]

**Additional file**

**Salicylic Acid Enhances Cell Growth, Fatty Acid and Astaxanthin Production in Heterotrophic *Chromochloris zofingiensis* without Reactive Oxygen Species Elevation**

Xinwei Zhang ^b†^, Zhao Zhang ^b, c†^, Yanmei Peng ^b^, Yushu Zhang ^b^, Qingyang Li ^a, *^, Dongzhe Sun ^a, *^

^a^ College of Life Sciences, Hebei Normal University, Shijiazhuang 050024, China.

^b^ School of Life Sciences, Hebei University, Baoding 071000, China.

^c^ Institute of Life Sciences and Green Development, Hebei University, Baoding 071000, China.

† Xinwei Zhang and Zhao Zhang contributed equally to this work.

* To whom corresponding should be addressed:

Tel.: +86-0311-80789730; fax: +86-0311-80789730

*E-mail address:* merdzsun@sina.com (Dongzhe Sun); qingyangli@hebtu.edu.cn

(Qingyang Li)

**Caption of Additional file figures**

Figure S1. Principal component analysis (PCA). To validate both the reproducibility of the triplicate parallel samples and the differences among different treatment groups, we conducted principal component analysis (PCA).

Figure S2. The differentially expressed genes detected by RT-qPCR at 24h, 48h and 72h.

**Caption of Additional file tables**

Table S1. Real-Time Quantitative PCR primer sequences. In order to verify the reliability of the transcriptome data, nine genes were randomly selected for which primers were designed.

Table S2. DEGs of different groups compared with control group cells at 24 h, 48 h and 72 h.

Table S3. Genes annotated in the network analysis and describe.


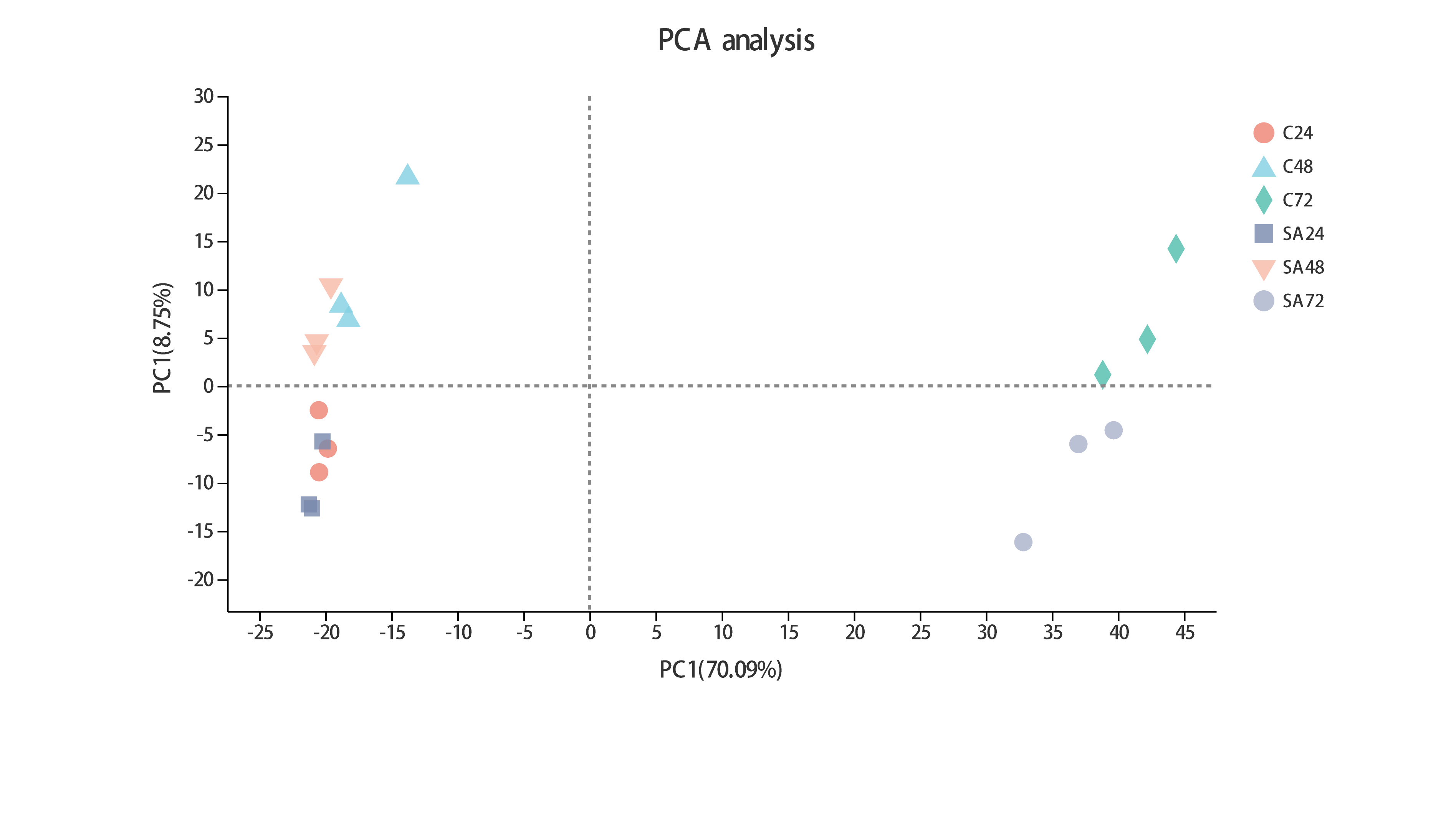


**Figure S1.** Principal component analysis (PCA).





**Figure S2.** The differentially expressed genes detected by RT-qPCR at 24h, 48h and 72h. Each sample was conducted with three biological replicates and the data points are represented as mean ± standard deviation (SD). Accase: acetyl-CoA carboxylase; AST: aspartate aminotransferase; AUH: methylglutaconyl-CoA hydratase; BKT: β-carotene-4-ketolase; CAT: catalase; fabG: 3-oxoacyl-[acyl-carrier protein] reductase; PDHA: pyruvate dehydrogenase E1 component alpha subunit; PDHC: pyruvate dehydrogenase E2 component; SOD2: superoxide dismutase.

**Table S1.** Real-Time Quantitative PCR primer sequences.

| **Gene** | **Forward (5’-3’)** | **Reverse (5’-3’)** |
| --- | --- | --- |
| CHZOF0001707 (Accase) | TGAGAATCCTGATAAAACATCCAGCAAACC | CACGCACCAGGTCAGTCACATTC |
| CHZOF0007785 (AST) | ACTTCGCCCAGTCTGTCCGTTTG | TGCCAGACAGCCCTGCCATAGAGAT |
| CHZOF0005754 (AUH) | TCTGTCTATGGCAGGGTGCTGAATG | GCAAATGCCTTCAATCCTTCCAGCC |
| CHZOF00011761 (BKT) | TATCCAAACCTCAAGCCGCACTC | ACCAACTGACTCTTCTTCGTGCC |
| CHZOF0005328 (CAT) | GCTGCCTCCCTATGTCCTTTTGC | GCACTTGGTTTGACTGACGCTGT |
| CHZOF0007885 (fabG) | GGTCGTCATAGTTACAGGAGCGTC | AGCCATACAGCACCTTCTGCCACC |
| CHZOF00014740 (PDHA) | GAGTCGTGTATGGCAGCGTGGGTTT | CCGCCAGTCACCTTGCGTAGTTT |
| CHZOF0001545 (PDHC) | TTTCGGGCTATGGCGAGGGTTTG | TGCCCAGGTTGGAGATGGTGAAGG |
| CHZOF0002713 (SOD2) | TGGGTTTCACTCTTCCTGACCTGCC | GCCAGATACCTGCTCCCAGTTGA |

**Table S2.** DEGs of different groups compared with control group cells at 24, 48 and 72h.

| **Group** | **Total DEG** | **Up** | **Down** |
| --- | --- | --- | --- |
| SA24_vs_C24 | 177 | 111 | 66 |
| SA48_vs_C48 | 366 | 105 | 261 |
| SA72_vs_C72 | 2962 | 1358 | 1604 |

**Table S3.** Genes annotated in the network analysis and describe.

| **Gene ID** | **Describe** |
| --- | --- |
| CHZOF00012193 | ABC transporter G family member 1; ATP-binding cassette, subfamily G (WHITE), member 2 |
| CHZOF00012760 | 3-oxoacyl-[acyl-carrier protein] reductase |
| CHZOF00013581 | nuclear migration nudc |
| CHZOF00014375 | component of RNA polymerase I core factor complex that acts as a GTF2B TFIIB-like factor and plays a key role in multiple steps during trancription initiation |
| CHZOF0002190 | amino acid transport and metabolism |
| CHZOF0003057 | integral component of membrane |
| CHZOF0003665 | methyl-CpG-binding domain protein 4 |
| CHZOF0005816 | rRNA binding |
| CHZOF0005893 | formate dehydrogenase |
| CHZOF0007490 | MFS transporter, SP family, solute carrier family 2 (myo-inositol transporter), member 13 |
| CHZOF0007940 | isopentenyl-diphosphate Delta-isomerase |
| CHZOF0008070 | histone deacetylase 6 |
| CHZOF0008278 | 4-aminobutyrate aminotransferase |
| CHZOF0008982 | solute carrier family 20 (sodium-dependent phosphate transporter) |
| CHZOF0009323 | solute carrier family 39 (zinc transporter), member 9 |
